# Supplementary material for: Complexome profiling on the Chlamydomonas lpa2 mutant reveals insights into PSII biogenesis and new PSII associated proteins
Source: J Exp Bot. 2021 Aug 26;73(1):245–62. doi: 10.1093/jxb/erab390 (PMC8730698; doi:10.1093/jxb/erab390)
Supplement: erab390_suppl_Supplementary_Dataset_S1 [file erab390_suppl_supplementary_dataset_s1.zip › Supplemental Dataset 1 - Excel List and all profiles/plots/LPA1_Cre10.g430150.html]

Trivial name: LPA1  
  
Euclidean distance: 13724.81  
Mean Intensity (WT): 2432.11  
Mean Intensity (Mut): 1889.30  
Distance: 5.64  
  
MapMan: not assigned.no ontology.pentatricopeptide (PPR) repeat-containing protein;PS.lightreaction.photosystem II.biogenesis  
  
p value of intensity sums Welch test: 0.2704
